# Exploring the independent association of employment status to cancer survivors’ health-related quality of life

**DOI:** 10.1186/s12955-023-02124-y

**Published:** 2023-05-11

**Authors:** Y. Andreu, C. Picazo, S. Murgui, A. Soto-Rubio, A. García-Conde, R. Romero

**Affiliations:** 1 Personality, Assessment and Psychological Treatments Department, Faculty of Psychology, University of Valencia, Valencia, Spain; 2 Psychology and Sociology Department, University of Zaragoza, Zaragoza, Spain; 3 Social Psychology Department, Faculty of Psychology, University of Valencia, Valencia, Spain; 4 Development and Education Psychology Department, Faculty of Psychology and Speech Therapy, University of Valencia, Valencia, Spain; 5 Psychology Unit - Valencian Institute of Oncology Foundation, Valencia, Spain

**Keywords:** Cancer survivors, Quality of life, Employment status, Age, Marital status, Survival phase, Cancer type

## Abstract

**Background:**

Having a job has been associated with better Health-Related Quality of Life (HRQOL) in cancer survivors. However, the sociodemographic and disease-related profiles characterizing the survivors being employed and those having better HRQOL largely overlap. The present study aims to discern the degree to which employment status is independently associated with cancer survivors’ HRQOL or if it mainly reflects the impact of other sociodemographic and cancer-related variables.

**Methods:**

Cross-sectional study on a heterogeneous sample of 772 working-age survivors of adult-onset cancer. An instrument specifically designed to assess HRQOL in cancer survivors and Multivariate Variance Analysis (MANOVA) were used.

**Results:**

Survival phase, cancer type, and employment status showed the main effects on cancer survivors’ HRQOL. In particular, being employed (vs unemployed) had the greatest positive association with HRQOL, affecting ten of the twelve HRQOL domains considered. Also, interaction effects highlighted the role of age (younger) and marital status (single) as risk factors for a greater negative impact of variables affecting the survivor’s HRQOL.

**Conclusions:**

The application of a multivariate methodology sheds new light on two relevant issues for the cancer survivor’s HRQOL: (i) the existence of differences between diagnostic groups that are not attributed to other variables such as sex, and (ii) the important and independent role that employment status plays. Comprehensive cancer survivorship care should focus more on high-risk groups and include having a job as an essential aspect to consider and prompt. The fact that the employment status is susceptible to change represents a valuable opportunity to care for the wellbeing of this population.

## Introduction

Advances in cancer treatment have made it possible to witness a steady increase in cancer survival for the last few decades. This improvement in cancer survival brings new hope for cancer patients and new challenges in cancer survivors’ attention and the care of their Health-Related Quality of Life (HRQOL). In this context, studying cancer’s long-term effects and their potential modulators is of pivotal relevance [1–3].

Cancer survivors often suffer from late and long-term physical effects such as pain, fatigue, and cognitive impairment (short-term memory, verbal expression, and spatial skills) as well as psychosocial problems related to fear of recurrence and difficulties in undertaking social, family and work roles [4–10]. Thus, even after several years of primary treatment completion, the HRQOL of cancer survivors might still be affected [8, 11]. Although results are still inconclusive and present limitations [12, 13], the literature points to the following as moderating variables of the impact of cancer on the survivor’s HRQOL: type and number of strategies included in the primary treatment [14, 15], age [16, 17], gender [2, 15], marital status [18, 19], education level [17, 18], and unemployment [19, 20].

Employment status deserves special attention given the central role that employment currently plays in people’s lives worldwide, especially in industrialised countries, where an average adult invests more than two-thirds of their time in their work [21, 22]. From an extrinsic or instrumental perspective, attempts have been made to explain the centrality of work in that it constitutes a resource for economic and material security [23]. However, a second theoretical approach emphasizes an intrinsic perspective, in which work is essential because it guarantees socio-psychological needs, fostering personal identity, self-esteem, status and a sense of success [21, 23]. Thus, employment is the main way of obtaining the economic resources needed to live and also a key tool that facilitates inclusion in social life [24] and promotes well-being.

In line with the second theoretical formulation, there are numerous benefits for cancer survivors who continue working. It allows cancer survivors to restore a sense of normality, identity and living conditions, provides social support and could even be considered a healthy distraction [25–28]. Return to work represents, in short, returning to the daily life from which the patients were temporarily excluded because of the cancer diagnosis and treatment. Thus, it is a significant factor affecting their QOL through the restoration of interpersonal relationships and social status [6, 29]. Several studies show that unemployed cancer survivors or those with more significant work-related impairments experience greater long-term psychological distress and worse QOL compared with those with a job and those who experience minor work-related impairments [19, 29, 30]. As with other workers, people with cancer use employment as a social and economic resource, but it also has a special significance because it allows them to gain confidence in their health and social status [31].

However, the data indicate that about half of those cancer survivors who are of working age [32] suffer a negative impact of cancer and its treatment that interfere with their ability to work [6, 33–35]. The side effects of cancer treatment can make it challenging to keep a job, forcing the individual to reduce their workload, take a break from work, or directly quit for good their jobs [36–38]. According to a recent meta-analysis [39], the cancer survivor’s ability to work continues to be significantly negatively impacted 6 years after diagnosis, on average. Furthermore, unemployment rates are significantly higher for cancer survivors than for the general population [35]. Cancer survivors are approximately 1.4 times more likely to be unemployed than people without a history of cancer, and roughly 25% of cancer survivors will not have returned to work 2 years after diagnosis [35]. In Spain, the scarce results available on this topic point out that cancer leaves roughly 25,000 survivors at risk of social exclusion each year, which accounts for almost a third (27.7%) of all diagnoses in the working population [40]. While around 53% of working-age cancer patients in Spain survive more than 5 years after diagnosis, only 45% of them will return to work [31, 40].

Although evidence so far supports the relevance of employment status in the cancer survivor’s HRQOL, caution must be taken when concluding that it is one objective outcome of their HRQOL [41]. In this context, it would be advisable to analyse the role of the employment status as independently associated with the HRQOL [33]. The type of work (physically or cognitively demands or job flexibility) also relates to employment status after cancer [6, 23, 29, 32]. In addition, the most frequently cited variables predicting employment status after cancer include sociodemographic factors such as gender [26], age [42], marital status [43], education level [44, 45], and chemotherapy [27]. Note that the variables predicting employment status overlap those that modulate HRQOL. Thus, it is necessary to discern whether employment status is independently associated with HRQOL in cancer survivors or if its role mainly reflects the impact of other sociodemographic and cancer-related variables.

In light of the above, the present study aims to overcome some of the limitations in the literature on the link between employment status and cancer survivors’ HRQOL. In particular, it explores the independent association between the two variables in a heterogeneous sample of survivors of adult-onset cancer with a disease-free status. It does so by using an instrument specifically designed to assess HRQOL in cancer survivors. Finally, the present study also analyses the independent association of employment status with cancer survivors’ HRQOL by controlling the effect of other sociodemographic variables such as age, gender, marital status, education level, and disease-related variables such as cancer type, primary treatment strategies and time elapsed since the end of primary treatment.

## Method

### Participants and procedure

This cross-sectional study is part of a research project on HRQOL and unmet psychosocial needs in adult oncology survivors in Spain, and it was approved by the Ethics Committee of the participating medical institutions and cancer patient associations; specifically, by the Research Ethics Committee of the Valencian Institute of Oncology Foundation (FIVO). Inclusion criteria for the general sample of participants were: i) to have been diagnosed with adult cancer; ii) to present no evidence of disease; and iii) to have completed primary treatment with curative intent (surgery, radiotherapy and chemotherapy) at least one month before the time of the study (time frame of reference explored by QLACS). Of the total number of survivors fulfilling inclusion criteria (*N* = 1862), the present study focuses exclusively on working-age cancer survivors. Therefore, data from 772 participants are analysed, all of them agreed to participate and provided written consent. A psychologist carried out the face-to-face assessment during one of the survivors’ visits to the health centres.

Participants’ age ranged from 22 to 64 years (M = 52.1; SD = 8.7), with a majority being over 45 years of age (79.7%). Most of them were women (68.9%), married or living with a partner (70.3%), did not have a university education (58.2%) and were employed (55.3%). The distribution of cancer type was breast (48.2%), colorectal (9.8%), prostate (9.8%), haematological (8.3%), head and neck (7.5%), gynaecological (6.7%), melanoma (4.9%), and multiple (4.7%).

Regarding primary treatment with curative intent, a substantial proportion of the participants (44.8%) had received combined treatment with radiotherapy and chemotherapy (with or without surgery); 38.5% had received local treatment, and 16.7% had received systemic treatment (with or without surgery). Lastly, the average length of time elapsed after the completion of primary treatment was 4.3 years (range: 1 month—30 years).

Concerning time of survival, the study follows the proposal of Stanton et al. [44] that distinguishes three survival phases. In this way, 20.3% of participants had completed primary treatment in the previous 12 months (re-entry survivorship phase, RES); 32.9% had completed it at least 5 years before the moment of interview (long-term survivorship phase, LTS), and 46.8% had exceeded 12 months after primary treatment but had not yet reached 5 years (early survivorship phase, ES) (Table 1).

**Table 1:** Characteristics of the participants

		**Total *N* = 772**	**Employed *n* = 427**	**Unemployed /Early retired *n* = 345**	**Chi**^**2**^
		**n (%)**	**n (%)**	**n (%)**	
Age (mean: 52.4; SD = 8.5; Range = 22–64)	≤ 45 years	157 (20.3)	116 (27.2)	41 (11.9)	27.51***
	46–64 years	615 (79.7)	311 (72.8)	304 (88.1)	
Gender	Female	532 (68.9)	314 (73.5)	218 (63.2)	9.54**
	Male	240 (31.1)	113 (26.5)	127 (36.8)	
Marital status	With Partner	543 (70.3)	302 (70.7)	241 (69.9)	.07
	Single	229 (29.7)	125 (29.3)	104 (30.1)	
Qualification	Higher education	304 (40.4)	230 (55.2)	74 (22.0)	84.85***
	No Higher education	449 (58.2)	187 (44.8)	262 (78.0)	
Cancer type	Breast	372 (48.2)	224 (52.5)	148 (42.9)	31.38***
	Prostate	76 (9.8)	38 (8.9)	38 (11.0)	
	Colorectal	76 (9.8)	33 (7.7)	43 (12.5)	
	Hematologic	64 (8.3)	37 (8.7)	27 (7.8)	
	Head & neck	58 (7.5)	20 (4.7)	38 (11.0)	
	Gynaecologic	52 (6.7)	35 (8.2)	17 (4.9)	
	Melanoma	38 (4.9)	27 (6.3)	11 (3.2)	
	Multiple	36 (4.7)	13 (3.0)	23 (6.7)	
Primary treatment	S, RT, or S + RT	297 (38.5)	174 (40.7)	123 (35.7)	2.56
	S, CT, or S + CT	129 (16.7)	72 (16.9)	57 (16.5)	
	S + CT + RT	346 (44.8)	181 (42.4)	165 (47.8)	
Survival phase	RES	157 (20.3)	80 (18.7)	77 (22.3)	2.96
	EH	361 (46.8)	211 (49.4)	150 (43.5)	
	LTS	254 (32.9)	136 (31.9)	118 (34.2)	

Primary treatment: *S* Surgery, *RT* Radiotherapy, *CT* Chemotherapy; Survival phase: *RES* Re-entry survivorship (≤ 12 months), *EH* Early survivorship (13–59 months), *LTS* Long-term survivorship (≥ 5 years)

^*^p < .05

^**^p < .01

^***^p < .001

### Instruments

#### Health-related QOL (HRQOL)

This variable was assessed with the Quality of Life in Adult Cancer Survivors (QLACS) scale [45], specifically with the Spanish version of Escobar and col. [46]. This scale comprises 47 items concerning twelve domains: negative feelings, positive feelings, cognitive problems, physical pain, problems with sexual functioning, fatigue, social avoidance, financial problems, family-related distress, appearance concerns, distress over recurrence, and cancer benefits. Moreover, each domain consists of 4 items except for family-related distress, with only three items (the resulting score from the latter is multiplied by 1.33 to be comparable with the other domains).

The period assessed with the QLACS focuses on the previous month, using a seven-point Likert scale (1 = never trough 7 = always) with higher scores indicating lower HRQOL (except for positive feelings and benefits of cancer). Previous results with the Spanish version support QLACS’ good psychometric properties [12]. The reliability indices obtained in this study were satisfactory (see Table 2). It should be noted that α = 0.67 for the distress-recurrence variable is an acceptable value of internal consistency since this scale has less than 10 items [47].

**Table 2:** Means, standard deviations, Cronbach’s alpha and Pearson correlations among the HRQOL domains

	M	SD	1	2	3	4	5	6	7	8	9	10	11	12
1. Negative feelings	12.75	5,06	.79											
2. Positive feelings	20.29	5,60	-,600**	.87										
3. Cognitive problems	11.41	6,22	,604**	-,399**	.83									
4. Sexual problems	12.50	6,62	,494**	-,399**	,428**	.84								
5. Pain	11.34	6,22	,613**	-,453**	,509**	,440**	.87							
6. Fatigue	12.88	5,84	,619**	-,509**	,601**	,531**	,697**	.89						
7. Social avoidance	8.60	5,18	,557**	-,552**	,460**	,453**	,502**	,477**	.90					
8. Appearance concerns	10.52	6,50	,398**	-,315**	,370**	,373**	,401**	,370**	,357**	.79				
9. Financial problems	7.47	5,30	,270**	-,225**	,428**	,237**	,426**	,349**	,258**	,395**	.76			
10. Distress-recurrence	14.42	6,60	,425**	-,291**	,324**	,307**	,365**	,308**	,299**	,457**	,282**	.67		
11. Distress-family	13.06	5,87	,229**	-,089*	,144**	,111**	,249**	,172**	,104**	,225**	,184**	,466**	.83	
12. Benefits of cancer	18.33	6,88	-,193**	,360**	-,090*	-,147**	-,083*	-,156**	-,204**	,019	,013	,103**	,167**	.86

^*^p ≤ .05, ^**^p ≤ .01, ^***^p ≤ .001. Significant differences

### Statistical analysis

Descriptive statistics were calculated to summarise sociodemographic, cancer-related and psychosocial data. A Multivariate Variance analysis (MANOVA), including all other sociodemographic and illness-related variables assessed as independent variables, was applied to the HRQOL domains. Due to the numerous independent variables, analyses were limited to the main effect and second-order interactions. Because of the different groups’ sizes, Pillai’s trace (V) was used to evaluate the multivariate significant overall differences. Follow-up univariate F tests were conducted, and significant results on the univariate tests were followed with Bonferroni’s comparisons between all possible pairs of means. The statistical significance level for analyses was *p* < 0.05. Statistical analysis was performed using IBM SPSS Statistics, version 22.0.

## Results

Descriptive data on sociodemographic and disease-related variables for the total sample and for the subgroups established according to employment status are shown in Table 1. Descriptive and correlational data for QLACS domains are shown in Table 2.

The results of the MANOVA showed a significant main effect for cancer type (V = 0.18, F(84, 4186) = 1.34, *p* < 0.05), time elapsed since the end of primary treatment (F(24, 1186) = 1.54, *p* < 0.05), and employment status (F(12, 592) = 5.25, *p* < 0.001) (See Table 3).

**Table 3:** MANOVA factorial (2^a^ × 8^b^ × 3^c^ × 2^d^ × 3^e^ × 2^f^ × 2^ g^ × 2^ h^) domains of HRQOL^*^

Source of variation	V	F	df between	df error
(A) Employment status	.096	5.250***	12	592
(B) Cancer type	.184	1.342*	84	4186
(C) Survival phase	.060	1.541*	24	1186
(D) Gender	.029	1.465	12	592
(E) Primary treatments	.051	1.298	24	1186
(F) Qualification	.024	1.207	12	592
(G) Age	.024	1.201	12	592
(H) Marital status	.015	.751	12	592
(A x B) employment status *Cancer type	.166	1.212	84	4186
(A x C) employment status * Survival phase	.040	.996	24	1186
(A x D) employment status * Gender	.011	.527	12	592
(A x E) employment status * Primary treatment	.047	1.179	24	1186
(A x F) employment status * Qualification	.016	.818	12	592
A x G) employment status * Age	.043	2.235**	12	592
(A x H) employment status * Marital status	.043	2.236**	12	592
(B x C) Cancer type * Survival phase	.301	1.109	168	7236
(B x D) Cancer type * Gender	.173	1.262	84	4186
(B x E) Cancer type * Primary treatment	.268	1.058	156	7236
(B x F) Cancer type * Qualification	.140	1.016	84,	4186
(B x G) Cancer type * Age	.233	1.718***	84	4186
(B x H) Cancer type * Marital status	.177	1.296*	84	4186
(C x D) Survival phase * Gender	.038	.967	24	1186
(C x E) Survival phase * Primary treatment	.099	1.255	48	2380
(C x F) Survival phase * Qualification	.034	.853	24	1186
(C x G) Survival phase * Age	.076	1.962**	24	1186
(C x H) Survival phase * Marital status	.058	1.466	24	1186
(D x E) Gender * Primary treatment	.041	1.023	24	1186
(D x F) Gender * Qualification	.028	1.430	12	592
(D x G) Gender * Age	.017	.869	12	592
(D x H) Gender * Marital status	.019	.960	12	592
(E x F) Primary treatment * Qualification	.030	.752	24	1186
(E x G) Primary treatment * Age	.060	1.540*	24	1186
(E x H) Primary treatment * Marital status	.035	.889	24	1186
(F x G) Age * Marital status	.023	1.182	12	592
(F x H) Age * Qualification	.015	.771	12	592
(G x H) Qualification * Marital status	.013	.651	12	592

*V* Pillai’s Trace value, *F* Fisher’s F value, *df* degrees of freedom

^*^p < .05

^**^p < .01

^***^p < .001

The univariate analysis (See Table 4) showed that longer survival time (differentiating among re-entry survivorship (RES), early survivorship (ES), and long-term survivorship (LTS) was associated with less fatigue (F(2, 603) = 4.48, *p* < 0.05) and fewer sexual problems (F(2, 603) = 3.65, *p* < 0.05) (although there were no differences between the groups).

**Table 4:** Means, standard deviations (in brackets), F values, and post hoc Bonferroni procedure for employment status groups and time since primary treatment across survivors and HRQOL measures

**Source of variation**	**Employment status**			**Survival phase**			
	**Employed**	**Unemployed**	**F**	**Re-entry phase**	**Early survival**	**Long survival**	**F**
Negative feelings	12,04 (4,77)	13,63 (5,27)	19,467***	12,69 (4,82)	12,90 (5,17)	12,57 (5,06)	1,675
Positive feelings	21,24 (5,22)	19,11 (5,85)	22,319***	19,89 (5,69)	20,40 (5,52)	20,38 (5,69)	.051
Cognitive problems	10,63 (5,21)	12,39 (5,70)	14,733***	10,90 (5,34)	11,59 (5,69)	11,47 (5,32)	2.378
Sexual problems	11,69 (6,39)	13,51 (6,77)	11,020**	12,70 (6,95)	12,89 (6,80)	11,84 (6,11)	3.647*
Pain	9,77 (5,12)	13,26 (6,88)	25,996***	11,79 (5,65)	11,54 (6,43)	10,78 (6,24)	1.521
Fatigue	11,41 (5,31)	14,71 (5,96)	30,357***	13,77 (5,75)^a^	13,02 (5,87)^a^	12,13 (5,78)^b^	4.482*
Social avoidance	8,09 (4,72)	9,23 (5,64)	9,796**	8,36 (5,05)	8,78 (5,37)	8,49 (4,99)	1.711
Appearance concerns	10,33 (6,29)	10,76 (6,75)	17,543***	10,62 (6,35)	10,42 (6,47)	10,60 (6,65)	1.032
Financial problems	6,46 (4,45)	8,72 (5,97)	19,456***	7,15 (5,09)	7,88 (5,85)	7,08 (4,55)	2.302
Distress-recurrence	14,27 (6,50)	14,61 (6,75)	1,022	13,87 (6,43)	14,36 (6,55)	14,86 (6,80)	.090
Distress-family	12,88 (5,66)	13,29 (6,13)	2,274	13,01 (5,93)	12,86 (5,86)	13,40 (5,88)	1.601
Benefits of cancer	19,15 (6,57)	17,31 (7,13)	6,047*	17,37 (7,20)	18,06 (6,99)	19,31 (6,43)	1.987

*F* Fisher’s F value

^*^p < .05

^**^p < .01

^***^p < .001

The superscripts ^a^ and ^b^ indicate differences between the subgroups in the direction a > b. On sexual problems domain differences between groups are not significant

Some types of cancer emerged as risk factors for increased vulnerability in specific domains of the HRQOL (See Table 5). Hematologic and breast cancer survivors were more affected by cognitive problems than the rest of cancer the cancer type subgroups (F(7, 603) = 2.16,* p* < 0.05). Appearance concerns (F(7, 603) = 3.43, *p* < 0.01) was most prominent in hematologic, breast and gynecologic cancer survivors than in the rest of the cancer type subgroups. Financial problems (F(7, 603) = 2.63, *p* < 0.05) were mainly present in hematologic, multiple, and head/neck cancer survivors. Family-related distress (F(7, 603) = 2.25, *p* < 0.05) was higher for multiple cancer survivors than for the rest of cancer type subgroups. Finally, distress over recurrence (F(7, 603) = 2.43, *p* < 0.05) was higher in multiple, hematologic, breast, and gynecologic cancer survivors than in the rest of the cancer type subgroups. In all cases, prostate cancer survivors were less affected than the rest of the cancer type subgroups.

**Table 5:** Means, standard deviations (in brackets), F values, and post hoc Bonferroni procedure for treatment type groups and HRQOL measures

Source of variation	Breast	Prostate	Colorectal	Hematologic	Head & neck	Gynaecologic	Melanoma	Multiple	F
Negative feelings	13,01 (4,96)	12,12 (5,08)	12,09 (5,23)	13,80 (5,27)	11,79 (4,93)	13,12 (5,08)	11,37 (5,02)	13,50 (5,23)	1.461
Positive feelings	20,09 (5,66)	20,57 (5,43)	20,37 (5,99)	19,19 (4,99)	21,24 (5,77)	20,46 (5,47)	21,34 (5,75)	20,58 (5,47)	1.166
Cognitive problems	12,24 (5,59)^b, e^	9,61 (5,02)^a, d^	11,01 (5,42)	12,97 (5,27)^b, e^	9,50 (4,75)^a, d^	10,08 (5,19)	9,47 (5,22)	11,94 (5,56)	2.159*
Sexual problems	12,90 (6,60)	14,75 (6,80)	11,21 (6,11)	13,51 (6,91)	9,34 (5,07)	13,71 (6,91)	9,00 (5,05)	11,81 (7,13)	.781
Pain	12,07 (6,02)	8,67 (5,59)	10,47 (6,26)	12,10 (6,62)	11,33 (6,15)	12,13 (7,40)	8,03 (4,24)	12,39 (6,46)	1. 818
Fatigue	13,44 (5,66)	11,09 (6,03)	12,75 (6,18)	13,62 (5,83)	11,43 (4,88)	13,31 (6,75)	10,39 (5,71)	14,23 (5,19)	1.860
Social avoidance	8,43 (4,98)	8,63 (6,19)	8,84 (5,81)	10,46 (5,34)	7,72 (4,87)	8,56 (4,72)	8,29 (4,57)	8,42 (4,59)	1.368
Appearance concerns	11,98 (6,80)^b, c, e, g^	5,85 (3,59)^a, d, f, h,^	8,75 (5,11)^a, d^	13,97 (6,57)^b, c, e, g^	7,66 (4,21)^a, d, f^	11,63 (6,95)^b, e, g^	6,81 (3,45)^a, d, f^	9,86 (6,08)^b^	3.425**
Financial problems	7,46 (5,46)	5,96 (3,19)^d, e, h^	7,10 (5,52)	9,23 (5,76)^b, g^	8,52 (5,57)^b^	7,42 (5,46)	5,58 (2,88)^d^	8,92 (6,11)^b^	2.634*
Distress-recurrence	14,96 (6,66)^b^	11,16 (5,46)^a, d^	13,59 (6,13)^b,^	15,77 (6,42)	13,07 (6,41)	15,29 (7,37)^b^	14,21 (6,34)	16,39 (6,75)^b^	2.430*
Distress-family	13,18 (5,87)	11,42 (6,01)^h^	14,16 (5,13)	12,48 (5,63)	12,48 (6,21)	12,83 (6,70)	12,66 (5,55)	15,75 (5,06)^b^	2.252*
Benefits of cancer	18,88 (6,66)	14,37 (7,56)	19,18 (6,45)	19,23 (6,15)	18,22 (7,25)	19,42 (6,14)	17,53 (7,15)	17,17 (7,36)	.863

*F* Fisher’s F value

^*^p < .05

^**^p < .01

^***^p < .001

For each domain of HRQOL, the superscripts indicate significant differences with respect to ^a^: breast; ^b^: prostate; ^c^: colorectal; ^d^: hematologic; ^e^: head & neck; ^f^: gynecologic; ^g^: melanoma; ^h^: multiple

Regarding employment status, being unemployed was associated with worse HRQOL in all domains except for family-related distress and distress over recurrence (See Table 4).

Interaction effects (See Tables 6, 7 and 8) showed different deterioration by age in several domains of HRQOL as a function of survival phase (F(24, 1186) = 1.96, *p* < 0.01), employment status (F(12, 592) = 2.34, *p* < 0.01), cancer type (F(84, 4186) = 1.72, *p* < 0.001), and primary treatment with chemotherapy (F(24, 4186) = 1.54, *p* < 0.05). The results of univariate analyses showed that survivors ≤ 45 years in the early survival phase presented more cognitive and sexual problems than survivors ≤ 45 years in the long survival phase and more negative feelings than survivors aged 46–64 years at any phase. Likewise, unemployed survivors ≤ 45 years were the subgroup with the greatest deterioration in positive feelings, financial problems, sexual problems and concern about appearance. Prostate cancer survivors ≤ 45 years reported (i) more pain than multiple diagnosis survivors ≤ 45 years and cancer survivors of prostate, colorectal, head and neck, and melanoma aged 46–64 years; and (ii) more fatigue than prostate survivors aged 46–64 years and melanoma survivors ≤ 45 years. Likewise, colorectal and multiple cancers survivors ≤ 45 years experienced greater distress-family than prostate and gynecologic cancer survivors ≤ 45 years. Finally, colorectal cancer survivors aged 46–64 years experienced greater distress-family than gynecologic cancer survivors ≤ 45 years. Bivariate analyses were not significant with respect to the primary treatment.

**Table 6:** Means, standard deviations (in brackets), and post hoc Bonferroni procedure for interaction employment status and age (upper level), and interaction employment status and marital status

	**Employed**	**Unemployed**	**Employed**	**Unemployed**	**Employed**	**Unemployed**	**Employed**	**Unemployed**
	**Positive feelings**		**Financial problems**		**Sexual problems**		**Appearance concerns**	
≤ 45 years	21.10^b^ (5.16)	14.20^a, c, d^ (4.50)	6.63^b, d^ (4.29)	11.27^a, c, d^ (7.28)	11.80^b^ (6.82)	16.85^a, c, d^ (7.45)	11.95^b, c, d^ (6.50)	17.03^a, c, d^ (7.47)
46–64 years	21.30^b, d^ (5.25)	19.50^c, b^ (5.90)	6.39^b, d^ (4.52)	8.37^a, b, c^ (5.70)	11.64^b, d^ (6.24)	13.05^b, c^ (6.56)	9.72^a, b, d^ (6.11)	9.92^a, b, c^ (6.19)
	**Negative feelings**		**Financial problems**		**Pain**		**Social avoidance**	
Single	11.45^f, h^ (4.48)	14.83^e, g, h^ (5.61)	7.30^f^ (4.55)	10.63^e, g, h^ 7.24)	9.33^f, h^ (4.62)	15.37^e, g, h^ (7.38)	7.89^f^ (4.42)	10.49^e, g, h^ (6.65)
With Partner	12.28^f^ (4.87)	13.11^f, e^ 5.04)	6.11^f, h^ (4.32)	7.89^f, g^ (5.12)	9.95^f, h^ (5.32)	12.36^f, g^ (6.46)	8.17^f^ (4.84)	8.70^f^ (5.07)

For each domain of HRQOL, the superscripts indicate significant differences with respect to ^a^: < 45 years employed; ^b^: < 45 years unemployed; ^c^: 46–64 years employed; ^d^: 46–64 years unemployed and ^e^: single employed; ^f^: single unemployed; ^g^: with partner employed; ^h^: with partner unemployed

**Table 7:** Means, standard deviations (in brackets), and post hoc Bonferroni procedure for interaction time since primary treatment and age

	** ≤ 45 years**			**46–64 years**		
	**Re-entry**	**Early survival**	**Long survival**	**Re-entry**	**Early survival**	**Long survival**
Negative feelings	13.19 (4.88)	15.00^c, d, e, f^ (5.40)	12.09^b^ (4.80)	12.53^b^ (4.81)	12.37^b^ (4.99)	12.68^b^ (5.12)
Cognitive problems	11.51 (5.87)	13.14^c, d^ (5.83)	10.53^b^ (4.85)	10.70^b^ (5.18)	11.20 (5.60)	11.68 (5.41)
Sexual problems	14.54^c^ (8.15)	14.12^c^ (7.32)	10.34^a, b^ (5.82)	12.11 (6.45)	12.57 (6.64)	12.16 (8.14)

For each domain of HRQOL, the superscripts indicate significant differences with respect to ^a^: < 45 years’ re-entry phase; ^b^: < 45 years’ early survival; ^c^: < 45 years long survival; ^d^: 46–64 years’ re-entry phase; ^e^: 46–64 years’ early survival; ^f^: 46–64 years long survival

**Table 8:** Means, standard deviations (in brackets), and post hoc Bonferroni procedure for interaction between cancer type and age, cancer type and marital status

	**Age**						**Marital Status**	
	** ≤ 45 years**	**46–64 years**	** ≤ 45 years**	**46–64 years**	** ≤ 45 years**	**46–64 years**	**Single**	**With Partner**
	**Pain**		**Fatigue**		**Distress-family**		**Financial problems**	
Breast	12.53^a^ (6.56)	11.95^a^ (5.87)	13.80 (5.97)	13.35^a^ (5.58)	13.81 (5.89)	13.01 (5.86)	9.34^a^ (6.90)	6.70^b^ (4.54)
Prostate	26.01^a, c^ (5.85)	8.20^b^ (4.87)	25.10^a, c^ (4.72)	10.72^b^ (5.65)	3.03^b^ (5.85)	11.65 (5.93)	7.00 (3.65)	5.73 (3.05)
Colorectal	10.75 (4.35)	10.45^d (^6.37)	12.25 (4.43)	12.77 (6.28)	19.25^a^ (3.50)	13.87 ^c^ (5.08)	9.21 (6.73)	5.70^b^ (4.06)
Haematological	11.19 (6.53)	12.85 (6.69)	13.14 (5.88)	14.00 (5.84)	12.75 (5.83)	12.26 (5.54)	9.96 (4.13)	8.78 (6.59)
Head and neck	12.17 (3.54)	11.23^d^ (6.40)	10.17 (4.54)	11.58 (4.94)	9.33 (2.58)	12.85 (6.41)	7.83 (5.71)	8.83 (5.54)
Gynaecological	10.95 (6.91)	13.00 (7.74)	11.77 (5.66)	14.43 (7.33)	10.09^b, d^ 6.12)	14.83 (6.47)	7.45 (6.20)	7.40 (4.95)
Melanoma	6.73^d^ (3.17)	8.56^d^ (4.55)	8.36^d^ (4.76)	11.22 (5.93)	11.91 (4.76)	12.96 (5.89)	8.38 (4.14)	4.83^b^ (1.93)
Multiple	9.75^d^ (6.50)	12.72^a^ (6.48)	12.25 (4.57)	14.48 (5.27)	19.25^a^ (2.87)	15.31 (5.13)	6.43 (4.76)	9.52^a^ (6.31)

For each domain of HRQOL, the superscripts ^a^ and ^b^ indicate differences between the subgroups in the direction a > b; likewise, the superscripts ^c^ and ^d^ indicate differences between the subgroups in the direction c > d

Interaction effects also showed different deterioration in HRQOL by marital status as a function of cancer type (F(84, 4186) = 1.30, *p* < 0.05) and employment status (F(12, 592) = 2.34, *p* < 0.01). Single and unemployed survivors showed more negative feelings, financial problems, pain and social avoidance than employed single survivors and survivors with partners (employed and unemployed). Furthermore, employed single survivors showed more negative feelings and pain than unemployed survivors with a partner. At the same time, unemployed survivors with a partner reported more financial problems and pain than employed survivors with a partner and more negative feelings than employed single survivors. Finally, single breast cancer survivors and multiple diagnosis survivors with a partner presented more financial problems than breast, colorectal and melanoma survivors with a partner.

## Discussion

The present study aimed to explore the role of employment status after the diagnosis and treatment of cancer on survivors’ HRQOL controlling the influence of several sociodemographic and disease-related variables. Our results supported the significant influence of the work status, the survival stage, and certain diagnoses on the cancer survivor´s HRQOL. In addition, the interaction effects highlighted the role of two sociodemographic variables as risk factors for a greater impact on the cancer survivor’s HRQOL: lower age and being single.

It has been noted that returning to work after the cancer experience would allow to retain a sense of normalcy, personal identity, and connectedness to others [25–28, 39], while the thought of not being able to have a job may represent a personal defeat that can lead to lasting difficulties [48]. Based on the results obtained, having a job has a positive impact on the domains of financial problems, sexual problems, concern about appearance, social avoidance, and negative and positive affectivity. This result is consistent with the authors’ previous findings regarding the higher prevalence of distress and unmet psychological, interpersonal, practical, and economic needs in unemployed colorectal cancer survivors [49].

In addition, having a job was also associated with better HRQOL in areas directly associated with common side effects of cancer treatment, such as pain and fatigue. The evidence suggests that, although seemingly counter-intuitive, rest is the wrong option to combat fatigue [50], highlighting, at the same time, the role of distraction in the perception of pain [51]. Given that worst symptoms also predict employment status, the specific relationship between these two variables is still to be elucidated. Nevertheless, it does not seem unreasonable to consider that bidirectionality is possible and that the activity and distraction of active working life [52] help reduce fatigue and pain perception. Therefore, supporting an intrinsic perspective [21, 23], data from this research show that having a job after cancer can be beneficial from a societal point of view and for the physical and mental health and the rehabilitation of the cancer survivor.

According to interactions effects, being unemployed is particularly associated with impaired HRQOL in single and ≤ 45 years’ survivors. Although previous research in this regard is inconsistent [53–56], our results underline the protective role of a partner, who acts as the primary source of social support. The existence of a partner buffers not only the financial impact of unemployment, but also its affective, social and even physical impact. Older age also plays a protective role in terms of the impact of unemployment on the cancer survivor HRQOL [7, 26]. In addition to the smaller financial impact of work status on survivors aged 46–64 years old, unemployment in this subgroup is also associated with lower concern for appearance and lower affectation in the emotional and sexual spheres.

Furthermore, our data revealed a greater economic impact of the cancer experience in multiple-cancer survivors with a partner and in breast cancer survivors that are single. It is not unlikely that repeated experiences with cancer will have a bigger impact on the family economy. In addition, the economic consequences of HRQOL deterioration due to prolonged (5–10 years) and frequent treatment with hormone therapy in breast cancer survivors [57] would be particularly visible in single breast cancer survivors.

According to different disease parameters, younger survivors are also a risk group to pay special attention to [19, 58]. Their HRQOL is particularly impaired when they have received chemotherapy as a primary treatment strategy as well as when they are in the early survival phase. Likewise, the better HRQOL usually associated with a diagnosis of prostate cancer [10, 19, 58, 59] does not seem to be presumed in survivors ≤ 45. Two key side effects of the cancer experience, pain and fatigue, are particularly relevant in this subgroup. Finally, the family-distress was found to be especially elevated in survivors ≤ 45 years with a multiple diagnosis as well as in those with colorectal cancer. It is true that the appearance of cancer at younger than normal ages as well as the appearance of more than one type of cancer in the same person are risk factors for the presence of hereditary genetic mutation [60–63]. However, it is also true that only 5–10% of total cancer cases are considered hereditary [61, 62]. It seems advisable, based on the obtained results, to approach the younger multiple and colorectal cancer survivor individually in order to clarify whether or not this concern is justified in his or her particular case. It may also be advisable to disseminate precise information on the subject by means of information campaigns. The increased social visibility given to breast cancer may be responsible for the fact that, despite sharing similar hereditary cancer figures to colorectal cancer [17, 19], the distress related to the risk of cancer among family members is not an equally prominent concern among survivors of breast cancer.

Although it was not the main objective of our study, the MANOVA results also revealed the role played by other variables frequently mentioned in the literature as predictors of HRQOL of cancer survivors. Despite the existing evidence regarding gender, educational level, and number/strategies of primary treatment [2, 12, 13, 15, 16, 18] as moderating variables of the cancer survivor’s HRQOL, these variables were not found to be independent predictors of the HRQOL in the age group analysed. In contrast, two disease-related variables did show an independent association with cancer survivor HRQOL: type of cancer and time since the end of primary treatment. Consistent with previous findings [24, 58], the results showed that the HRQOL of cancer survivors continues to be affected even when a long time has elapsed since the end of primary treatment. Also, although researches comparing HRQOL among survivors with different types of cancer are rare, the results of this study are consistent with most of the existing evidence [10, 19, 58, 63]. Hematologic, breast, and gynecologic cancer survivors experienced the greatest impact on their HRQOL; the least impact on HRQOL was experienced by prostate and melanoma cancer survivors, with the remaining cancer types occupying intermediate positions. Likewise, and given that the effect of cancer type on HRQOL remained significant after controlling for the effect of other sociodemographic and disease-related variables, our results support that differences in HRQOL between diagnostic subgroups are not due to other variables frequently associated with cancer type such as sex, age at diagnosis, and number/type of primary treatment strategies. It seems necessary to consider less obvious variables that also differentiate diagnostic groups (such as body area irradiated, chemotherapy protocol, maintenance treatment, etc.) to fully understand differences in HRQOL related to cancer type.

It is noteworthy that from the variables associated with the cancer survivor´s HRQOL, especially for those younger and single, only employment status is susceptible to modification. This susceptibility to modification is a valuable opportunity to care for the wellbeing of this population. Currently, the risk of unemployment for cancer survivors (where over 50% are of working age) is higher than for the general population [21, 35]. In addition, cancer survivors who return to work often report a loss of income due to a change in the workplace or their professional role, a decreased scope of work and even early retirement [35]. Thus, the ability to work, which is not only a function of one’s capacities (e.g. physical and mental abilities) but also of the job demands and resources [56], requires significant attention in the integral care of the cancer survivor. The social sector should play a complementary role to the health sector in improving the reintegration of cancer survivors into normal social roles and activities without discrimination, not only in Spain, where indeed these measures are pertinent and needed [64] but also in any country where the wellbeing of cancer survivors is trying to be preserved and promoted.

For all of the above, it is essential to acknowledge and address the concerns that are often expressed by cancer survivors relating to the workplace (e.g. disclosing their diagnosis), their ability to work, their physical appearance, and the difficulties when negotiating workplace accommodations with employers [24, 65]. Survivors also express a need to be guided and supported by health care professionals and vocational providers in order to have a job [7, 21, 49]. For Cancer survivors, having a job can also be facilitated by the willingness of employers to make job accommodations that mitigate the effects of cancer by maintaining a supportive work culture and environment. To this aim, raising awareness about the possibility, the need, the benefits and the importance of such accommodations is crucial, and also about the negative impact that the lack of them could signify for the cancer survivors, the work culture and environment, and lastly, the prosperity of the job entity. The significance of such adjustments is underlined by findings indicating that cancer survivors who receive workplace accommodations or whose jobs have more favourable employment protection policies have better employment outcomes [66]. In addition, a favourable psychosocial work environment has been shown to positively correlate with work skills [34, 67]. Thus, it is necessary to tackle integral policies and strategies that ensure access to employment and the appropriate adaptations in the workplace, all of which will safeguard the cancer survivors’ wellbeing and quality of life.

Current results regarding the effectiveness of RTW interventions point to multidisciplinary programmes (typically comprising a combination of psycho-educational, vocational, physical, medical or pharmacological interventions) as those associated with better employment outcomes [68], even though data based on randomised controlled trials does not support an improvement in employment rates of cancer survivors from targeted interventions [69]. A possible explanation for the disappointing results of such targeted interventions is that most of them are focused on the short-term [70].

Indeed, work limitations arising from the complex nature of the consequences of cancer and its treatment make the need for employment intervention plans to extend throughout cancer care and the superiority of multidimensional interventions unsurprising [71]. Thus, it has been pointed out that effective screening of survivors’ work-related limitations, concerns, and goals should be addressed throughout care [22]. In addition, health care professionals can play an essential role in the challenging process of having a job by providing the survivor with guidance, support, and functional and emotional assistance [72]. In this line, the European Commission’s Joint Action on Cancer Control [1] points out that psychosocial and vocational rehabilitation should take a person-centred approach and be supplied as part of a comprehensive care program essential for successful survival. Also, the National Comprehensive Cancer Network (NCCN) Survivorship Panel [65] has outlined a framework to aid clinicians in systematically addressing work-related concerns of cancer survivors after active disease treatment. This panel recommends that communication about a patient’s work and employment begin early in the course of decision-making about treatment and that work-related concerns should be regularly re-evaluated to provide appropriate support. The panel also recommends a multidisciplinary team approach that involves social work, primary care, physical therapy/occupational therapy, cancer rehabilitation and vocational counselling services. Therefore, advances in the development, implementation, and evaluation of survivorship care, including comprehensive rehabilitation services, are highly needed [64].

In sum, results from the present study underline the importance of employment status as closely related to the cancer survivor’s HRQOL, together with other variables related to the disease, such as type of cancer and time elapsed since the end of primary treatment, based on the data analysis from a large and heterogeneous sample of working-age cancer survivors. Notwithstanding the overall attention required by these variables, our results underline the need for special consideration of single survivors and younger survivors, since they both appear to be particularly vulnerable subgroups.

Strengths of this work are the large and heterogeneous sample by cancer type, the use of an HRQOL instrument specifically aimed at the cancer survivor population, and the application of the multivariate methodology that allows determining the variance in HRQOL explained by employment status (controlling for the variance in HRQOL linked to other sociodemographic and disease-related variables).

Despite its significant contributions, this study is not without limitations. For instance, this paper focused on the impact of employment versus unemployment on cancer survivors' HRQOL. Perhaps, other employment difficulties less visible than unemployment (such as underemployment, employment instability, absenteeism, presenteeism, decreased work productivity, and decreased worker wellbeing) [73] could also have been taken into consideration. Also, because of the cross-sectional design and the small size of some of the subgroups established for comparative purposes, the results’ interpretations should be made with caution. For example, the age range has been dichotomized in order to facilitate statistical analysis with more representative group sizes. Nevertheless, being closer to the age of retirement could be an important factor to consider more carefully in future research. Also, future research with longitudinal designs is needed, that study other variables that might be playing a role in the HRQOL such as rehabilitation programs that some cancer survivors may undergo for improving their QOL, as well as deepening into the variety of occupational difficulties that cancer survivors may face and, finally, identifying factors that hinder having a job in order to establish high-risk subgroups. Given that the care for employment outstands as an essential aspect of comprehensive cancer survivorship care, the early detection of such risk groups would allow for prioritising efforts regarding their employment status and contributing successfully to the quality of life of cancer survivors.

## Data Availability

The datasets generated during and/or analyzed during the current study are available from the corresponding author upon reasonable request.
